# Supplementary material for: D-Serine reduces the expression of the cytopathic genotoxin colibactin
Source: Microb Cell. 2023 Mar 6;10(3):63–77. doi: 10.15698/mic2023.03.793 (PMC9993432; doi:10.15698/mic2023.03.793)
Supplement: Supplementary file 1 [file mic-10-063-s01.pdf]

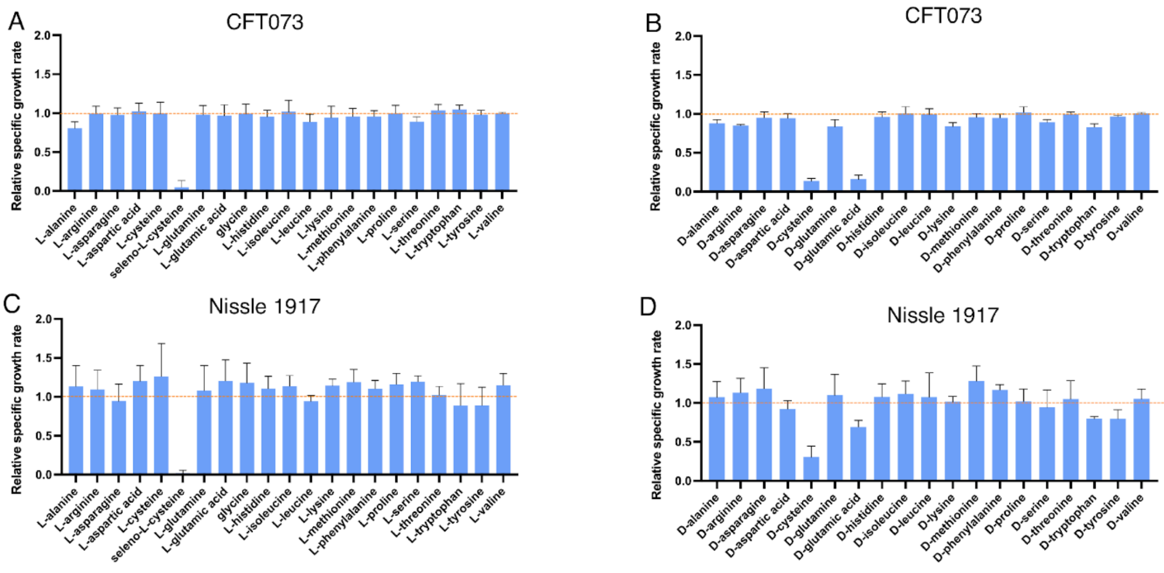

Supplemental Figure S1: Specific growth rate of *E. coli* CFT073 (A and B) and Nissle 1917 (C and D) in the presence of different amino acids. Bacteria grown in M9 media -/+ 1 mM of the stated amino acid and incubated at 37°C, 200 RPM. Optical density ($OD_{600\ nm}$) was recorded hourly, the specific growth rate ($\mu = \Delta lnOD600/\Delta t$) was calculated from the exponential period of growth, and data were expressed relative to the control (without any AA). Error bars denote the standard deviation calculated from the means of at least three independent biological replicates.

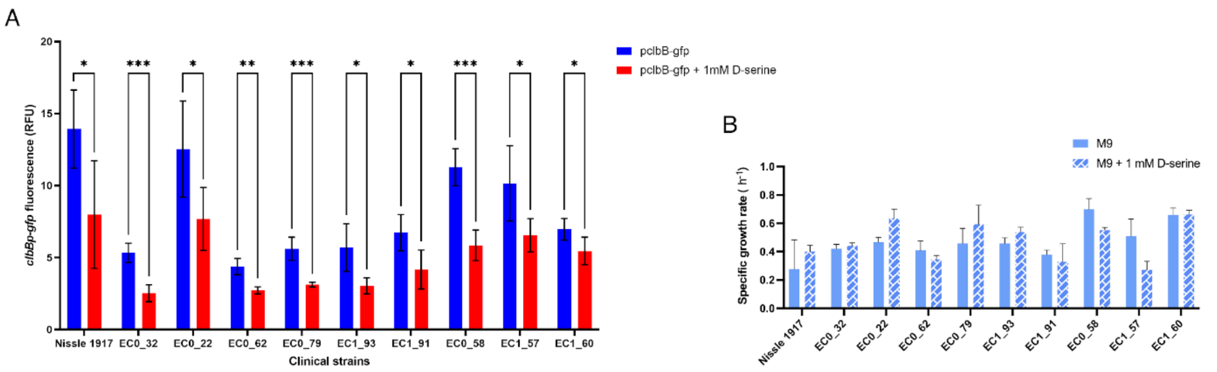

Supplemental Figure S2: Expression of colibactin and growth of clinical isolates of phylogroup B2 *E. coli* in the presence and absence of 1mM D-serine. (**A**) Clinical *E.coli* strains belonging to the B2 phylogroup were transformed with a reporter gene fusion (*pclbB:gfp*) to assess expression of genotoxic colibactin. Bacteria were grown in MEM-HEPES with and without 1mM D-serine and sampled at 4 hours following inoculation. Mean of relative fluorescence units (RFU = GFP/OD_{600}) for each strain were measured and plotted. Blue bars denote fluorescence of *pclbB:gfp* strains in the absence of D-serine; red bars denote fluorescence in the presence of D-serine. *E. coli* Nissle is included for comparative purposes. Error bars represent standard deviation. Statistical analysis was conducted via an unpaired Student's t-test. Asterisks (*) denote statistical significance levels: *: p-value < 0.05; **: p-value < 0.01, ***: p-value < 0.001. (**B**) Isolates were cultured in the presence and absence of 1 mM D-serine as indicated. Optical density ($OD_{600\ nm}$) was recorded hourly, and the specific growth rate ($\mu = \Delta lnOD600/\Delta t$) was calculated from the exponential period of growth. Data are from three biological replicates and are expressed as mean growth rate per hour with bars indicating standard deviation.

Supplemental Table S1. Fold changes and *P* values of *clb* genes in CFT073 determined by RNA-Seq.

		WT vs WT +D-Serine		Δ*dsdC* vs Δ*dsdC* +D-Serine	
**Feature ID**	**Name**	**Fold Change**	**FDR Corrected p- value**	**Fold Change**	**FDR Corrected p- value**
c2450	clbS	1.34	0.53	-1.38	7.00E-02
c2451	clbQ	-1.28	0.54	-1.22	4.10E-01
c2452	clbP	-1.28	0.56	-1.48	1.00E-02
c2453	clbO	-1.67	5.10E-03	-1.57	1.02E-03
c2455	clbN	-1.59	0.01	-1.78	6.42E-06
c2456	clbM	-1.74	3.01E-03	-2.47	3.59E-11
c2457	clbL	-1.88	1.33E-03	-2.19	1.60E-08
c2458	clbK	-1.84	6.87E-04	-1.99	9.00E-08
c2459	clbJ	-1.69	2.31E-03	-2.26	1.62E-10
c2460	clbI	-2.16	5.06E-06	-3.22	0.00E+00
c2463	clbH	-2.05	3.08E-05	-2.66	2.91E-13
c2464	clbG	-2.48	1.55E-05	-3.65	4.75E-19
c2465	clbF	-17.84	0.4	-36.67	2.97E-03
c2466	clbE	-3.06	0.5	-3.5	1.00E-01
c2467	clbD	-2.19	5.31E-03	-2.74	1.06E-07
c2468	clbC	-2.29	1.55E-05	-2.82	1.96E-12
c2470	clbB	-2.38	1.08E-07	-2.72	0
c2471	clbR	-22.11	0.4	-1.1	1
c2472	clbA	-1.81	1	1.75	7.80E-01

Supplemental Table S2. Relative gene expression and *P* values in CFT073 determined by RT-qPCR.

Amino Acid	Relative *clbB* Expression to No Amino Acid Control in CFT073	Positive Error	Negative Error	*P*-Value	Relative Fold Change
D-Alanine	0.44	0.32	0.19	0.019	2.28
D-Arginine	0.84	0.04	0.04	0.153	1.19
D-Asparagine	0.64	0.22	0.16	0.053	1.55
D-Aspartic Acid	0.80	0.05	0.05	0.120	1.25
D-Cysteine	0.11	0.04	0.03	0.009	9.11
D-Glutamic Acid	0.26	0.15	0.09	0.007	3.84
D-Glutamine	0.47	0.17	0.12	0.011	2.11
D-Histidine	0.71	0.14	0.12	0.068	1.41
D-Isoleucine	0.44	0.02	0.02	0.023	2.29
D-Leucine	0.69	0.24	0.18	0.077	1.44
D-Lysine	0.91	0.18	0.15	0.298	1.10
D-Methionine	1.11	0.55	0.37	0.301	1.11
D-Phenylalanine	0.96	0.59	0.36	0.480	1.05
D-Proline	0.66	0.22	0.17	0.059	1.51
D-Serine	0.32	0.2	0.12	0.006	3.12
D-Threonine	0.62	0.16	0.13	0.038	1.62
D-Tryptophan	0.39	0.12	0.09	0.013	2.54
D-Tyrosine	0.43	0.02	0.02	0.022	2.34
D-Valine	1.53	1.01	0.61	0.158	1.53
Glycine	1.09	0.15	0.13	0.316	0.91
L-Alanine	0.94	0.48	0.32	0.462	1.07
L-Arginine	0.98	1.06	0.51	0.385	1.02
L-Asparagine	1.29	0.46	0.34	0.159	1.29
L-Aspartic Acid	0.13	0.14	0.07	0.003	7.52
L-Cysteine	0.71	0.66	0.34	0.310	1.40
L-Glutamic Acid	0.92	0.13	0.11	0.261	1.09
L-Glutamine	1.01	0.12	0.1	0.493	0.99
L-Histidine	0.65	0.13	0.11	0.045	1.55
L-Isoleucine	0.11	0.02	0.01	0.007	8.84
L-Leucine	0.28	0.09	0.07	0.005	3.61
L-Lysine	5.33	11.83	3.67	0.162	5.33
L-Methionine	1.45	0.86	0.54	0.170	1.45
L-Phenylalanine	0.25	0.08	0.06	0.007	3.94
L-Proline	0.76	0.32	0.22	0.161	1.32
L-Selenocysteine	0.16	0.05	0.04	0.010	6.17
L-Serine	1.04	0.08	0.08	0.448	0.97
L-Threonine	0.93	0.72	0.41	0.490	1.08
L-Tryptophan	0.84	0.37	0.26	0.271	1.19
L-Tyrosine	0.82	0.19	0.15	0.158	1.23
L-Valine	0.37	0.28	0.16	0.009	2.67

Supplemental Table S3. Relative gene expression and *P* values in Nissle 1917 determined by RT-qPCR.

Amino Acid	Relative *clbB* Expression to No Amino Acid Control in Nissle 1917	Positive Error	Negative Error	*P*-Value	Relative Fold Change
D-Alanine	0.42	0.23	0.15	0.050	2.39
D-Arginine	0.74	0.42	0.27	0.214	1.35
D-Aspartic Acid	0.56	0.54	0.28	0.150	1.77
D-Cysteine	0.48	0.39	0.21	0.076	2.07
D-Glutamic Acid	0.58	0.03	0.03	0.086	1.73
D-Glutamine	0.32	0.01	0.01	0.043	3.08
D-Isoleucine	2.49	0.39	0.34	0.005	2.49
D-Lysine	0.62	0.61	0.31	0.188	1.61
D-Methionine	0.30	0.37	0.17	0.037	3.35
D-Serine	0.26	0.08	0.06	0.036	3.81
D-Tryptophan	0.65	0.09	0.08	0.108	1.54
D-Tyrosine	0.32	0.02	0.02	0.043	3.08
L-Histidine	0.52	0.15	0.12	0.069	1.93
L-Leucine	0.32	0.02	0.02	0.042	3.12
L-Phenylalanine	1.01	0.56	0.36	0.479	1.01
L-Selenocysteine	0.18	0.08	0.05	0.029	5.64
L-Threonine	0.76	0.68	0.36	0.346	1.32

Table S4. Strains and plasmids used in this study.

Strain/Plasmid	Description	Reference
CFT073	UPEC O6:K2:H1, from acute pyelonephritis	[80]
DH10B pBAC-*pks*	DH10B harbouring the bacterial artificial chromosome (BAC) bearing the complete *pks*-island (BAC-*pks*)	[81]
DC10B	Competent *E. coli* K12 cloning vector	[82]
Nissle 1917	Commensal O6:K5:H1, probiotic	Wall Lab Inventory
Δ*dsdC*	Nissle 1917 *dsdC* deletion mutant	This study
EC0_32	*E. coli* O1:H7, isolated from catheter	[47]
EC0_22	*E. coli* O6:H1, isolated from catheter	[47]
EC0_62	*E. coli* O2:H7, isolated from catheter	[47]
EC0_79	*E. coli* O6:H1, isolated from catheter	[47]
EC1_93	*E. coli* O6:H31, isolated from catheter	[47]
EC1_91	*E. coli* O18:H1, isolated from catheter	[47]
EC0_58	*E. coli* O18:H7, isolated from catheter	[47]
EC1_57	*E. coli* O18:H31, isolated from catheter	[47]
EC1_60	*E. coli* O22:H1, isolated from catheter	[47]
pKD46	Lambda red helper plasmid, 30ºC temperature sensitive origin, arabinose inducible, ampR	[83]
pCP20	FRT recombinase plasmid for excision of chlR from mutants, ampR	[83]
p*clbB*::GFP	CFT073 *clbB* promoter region-GFP fusion reporter in pAJR70, chlR	This study

Supplemental Table S5. Oligonucleotide sequences used in this study.

Primer	Sequence (5'-3')	Use
dsdC.red.Fw	ACATCTAAAAATGAAGGTGAATTGAGATATGGTTCACT TATAGCTCACCTTAGTGTAGGCTGGAGCTGCTTC	Lamda red Nissle 1917 *dsdC* deletion cassette amplification
dsdC.red.Rev	TGACAAAACAATTCCCATATAAAAATTGCAATTTATAA AAGCCAACATACACATATGAATATCCTCCTTA	Lamda red Nissle 1917 *dsdC* deletion cassette amplification
dsdC Check Fw	CGCAGGCTGACAAACGATAA	*dsdC* deletion check
dsdC Check Rev	GCTCTCCAATATTCGACGCC	*dsdC* deletion check
PclbB-Fw	CCGGATCCGCTGTATCAATTCATACCCGCT	*CFT073 clbB* promoter-*gfp* fusion reporter
PclbB-Rev	CCGGTACCACGCGTGTCCATCTTATTACA	*CFT073 clbB* promoter-*gfp* fusion reporter
clbB.Q.Fw	TTGTCTCCGGATGTGTGTCA	CFT073 & Nissle 1917 *clbB* QPCR primer
clbB.Q.Rev	CACATCGTCAGCATAGCACC	CFT073 & Nissle 1917 *clbB* QPCR primer
gapA.Q.Fw	TTTCCGTGCTGCTCAGAAAC	CFT073 & Nissle 1917 *gapA* QPCR primer
gapA.Q.Rev	GGCCGTGAGTGGAGTCATAT	CFT073 & Nissle 1917 *gapA* QPCR primer

REFERENCES

47. Goswami C, Fox S, Holden M, Connor M, Leanord A, and Evans TJ (**2018**). Genetic analysis of invasive Escherichia coli in Scotland reveals determinants of healthcare-associated versus community-acquired infections. **Microb Genom** 4(6): e000190. doi: 10.1099/mgen.0.000190

80. Mobley HL, Green DM, Trifillis AL, Johnson DE, Chippendale GR, Lockatell CV, Jones BD, and Warren JW (**1990**). Pyelonephritogenic Escherichia coli and killing of cultured human renal proximal tubular epithelial cells: role of hemolysin in some strains. **Infect Immun** 58(5): 1281–1289. doi: 10.1128/iai.58.5.1281-1289.1990

81. Nougayrède J-P, Homburg S, Taieb F, Boury M, Brzuszkiewicz E, Gottschalk G, Buchrieser C, Hacker J, Dobrindt U, and Oswald E (**2006**). Escherichia coli induces DNA double-strand breaks in eukaryotic cells. **Science** 313(5788): 848–851. doi: 10.1126/science.1127059

82. Monk IR, Shah IM, Xu M, Tan M-W, and Foster TJ (**2012**). Transforming the Untransformable: Application of Direct Transformation To Manipulate Genetically Staphylococcus aureus and Staphylococcus epidermidis. **mBio** 3(2): e00277-11. doi: 10.1128/mBio.00277-11

83. Datsenko KA, and Wanner BL (**2000**). One-step inactivation of chromosomal genes in Escherichia coli K-12 using PCR products. **Proc Natl Acad Sci U S A** 97(12): 6640–6645. doi: 10.1073/pnas.120163297
